# Field Trapping *Bactrocera latifrons* (Diptera: Tephritidae) with Select Eugenol Analogs That Have Been Found to Attract Other ‘Non-Responsive’ Fruit Fly Species

**DOI:** 10.3390/insects9020050

**Published:** 2018-05-01

**Authors:** Grant T. McQuate, Jane E. Royer, Charmaine D. Sylva

**Affiliations:** 1 USDA-ARS, Daniel K. Inouye U.S. Pacific Basin Agricultural Research Center, 64 Nowelo Street, Hilo, HI 96720, USA; Charmaine.Sylva@ars.usda.gov; 2 Department of Agriculture and Fisheries, P.O. Box 267, Brisbane, QLD 4000, Australia; Jane.Royer@daf.qld.gov.au

**Keywords:** *Bactrocera latifrons*, dihydroeugenol, isoeugenol, methyl-isoeugenol, alpha-ionol, cade oil

## Abstract

*Bactrocera latifrons* (Hendel) (Diptera: Tephritidae) is a pest fruit fly species native to Oriental Asia which has invaded and established in Hawaii and Tanzania and has been recovered in detection trapping in California. It is largely non-responsive to the male lures cuelure and methyl eugenol. Alpha-ionol + cade oil is a moderately effective male *B. latifrons* attractant, but is not as attractive as cuelure or methyl eugenol are to other fruit fly species. An improved attractant is therefore desired. With the recent success in finding other non-responsive fruit fly species attracted to isoeugenol, methyl-isoeugenol, or dihydroeugenol in Australia and other countries, we wanted to assess whether *B. latifrons* might also respond to these “eugenol analogs.” Working with wild *B. latifrons* populations in Hawaii, we assessed the relative catch of *B. latifrons* in traps baited with the eugenol analogs with catch in traps baited with alpha-ionol, alpha-ionol + cade oil, or alpha-ionol + eugenol. Catch was significantly higher in traps baited with alpha-ionol + cade oil relative to traps with any of the other baits. There was, though, some male *B. latifrons* catch in traps baited with dihydroeugenol or isoeugenol but none in traps baited with methyl-isoeugenol.

## 1. Introduction

The Dipteran family Tephritidae includes over 4900 “fruit fly” species in more than 500 genera and are found in all world regions except Antarctica. Many of these species are pests that affect local food production and horticultural trade [1,2]. In the tribe Dacini (subfamily Dacinae), less than half of all species are responsive to the male lures methyl eugenol or cuelure [3]. These are effective attractants which have considerable value for monitoring, control, and taxonomy [4]. Dacini consists of the genera *Bactrocera, Dacus, Monacrostichus* and *Zeugodacus* [3,5,6,7] which are concentrated in two areas of the world: the Afrotropical Region and from Southeast Asia to Oceania [8]. Based on current available data, there are 932 described species in the tribe Dacini, including 461 *Bactrocera*, 273 *Dacus*, 196 *Zeugodacus,* and two *Monacrostichus* species [3]. Fifty-seven percent of these species do not respond to either methyl eugenol or cuelure (“non-responsive” species) or are only weakly attracted to them [3,5,6]. In a list of the *Bactrocera* spp. and *Dacus* spp. in Indomalaya, Wallacea and Australasia that are economically important, 22 species were listed as being responsive to cuelure, 15 species responsive to methyl eugenol and eight non-responsive species in the genus *Bactrocera*, and one cuelure responsive and one non-responsive species in the genus *Dacus* [6]. Lures are needed for non-responsive species to improve the ability to monitor and/or control field populations. Field testing of prospective new lures has been conducted in Australia and Papua New Guinea and resulted in discoveries of lure responses for some non- or weakly-responsive species. The most attractive novel lures identified in these studies were isoeugenol, methyl-isoeugenol, dihydroeugenol and zingerone [9,10,11].

*Bactrocera latifrons* (Hendel) is a polyphagous tephritid fruit fly species native to Oriental Asia [2]. It infests 59 hosts from 14 plant families but it is primarily a pest of Solanaceae and Cucurbitaceae [12,13]. It has invaded and established resident populations in Hawaii [14,15] and Tanzania [16] and has also been recovered in detection trapping in California [17]. *Bactrocera latifrons* is considered a non-responsive species, although is very weakly responsive to methyl eugenol [18]. In the list of the *Bactrocera* spp. and *Dacus* spp. in Indomalaya, Wallacea and Australasia that are economically important, *B. latifrons* was listed as a non-responsive species [6]. An alternative male lure for *B. latifrons*, alpha-ionol, was identified [19] and later found to be synergistically enhanced by the addition of cade oil [18,20]. Alpha-ionol + cade oil is the best male lure identified thus far for *B. latifrons*, but male response to it is not as strong as the response of other tephritid fruit fly species to cuelure or methyl eugenol. In a recent preliminary account of the fruit fly fauna of Timor-Leste, 16 *Bactrocera* spp. and one *Dacus* sp. were recovered in traps baited with either cuelure or methyl eugenol [21]. *Bactrocera latifrons* was recovered through fruit collections, but was not recovered in traps baited with alpha-ionol + cade oil. This points to the need to develop an improved attractant for *B. latifrons*, preferably one to which multiple species are attracted in order to minimize the numbers of lures needed to reliably detect the presence of tephritid fruit fly species of economic importance. With the recent success in finding some non-responsive fruit fly species attracted to isoeugenol, methyl-isoeugenol, and/or dihydroeugenol in Australia and Papua New Guinea [9,10], we wanted to assess whether *B. latifrons* might also respond to one or more of these “eugenol analogs”. We report herein results of field trials set up to assess such potential attractiveness.

## 2. Materials and Methods

### 2.1. Lures

The following sources were used for lures used in this trial: alpha-ionol (4-(2,6,6-trimethyl-2-cyclohexen-1-yl)-3-buten-2-ol, CAS 25312-34-9; 92% pure) (obtained from Bedoukian Research, Inc., Danbury, CT, USA); dihydroeugenol (2-methoxy-4-propylphenol, CAS 2785-87-7; ≥99% pure); eugenol (2-methoxy-4-(2-propenyl)phenol, CAS 97-53-0; 99% pure), isoeugenol (2-methoxy-4-propenylphenol, CAS 97-54-1; 99% pure), methyl-isoeugenol (1,2-dimethoxy-4-propenylbenzene, CAS 93-16-3; ≥98% pure) (all from Sigma-Aldrich, Castle Hill, New South Wales, Australia); and cade oil (CAS 8013-10-3, Penta Manufacturing, West Caldwell, NJ, USA). Cade oil, an essential oil produced by destructive distillation of *Juniperus oxycedrus* L. twigs, contains over 200 compounds, including at least four (eugenol, isoeugenol, dihydroeugenol and 2-methoxy-4-ethylphenol) which can synergize the attractiveness of alpha-ionol to male *B. latifrons.* These four compounds have approximate concentrations of 0.087%, 0.073%, 0.12% and 0.20%, respectively, in cade oil. In the present trial, we chose to only test one of these compounds (eugenol), as a pure compound with alpha-ionol, as this combination had been tested in previous field trials [18]. 

### 2.2. Study Site

The study site was in a cattle pasture, with extensive patches of turkeyberry (*Solanum torvum* Sw.), on the ocean-side of Loa Road in the vicinity of Pepeekeo, Hawaii (Hawaii Island). Turkeyberry is known to be a preferred host of *B. latifrons* [12,13,14,22,23]. One trap of each of the six treatments was set out in each of five patches (blocks). The coordinates of the middle patch were: Universal Transverse Mercator [UTM] grid [24]: Easting 0281761, Northing 2,194,920 m, Zone 05 Q, and was at 32 m elevation.

### 2.3. Bioassay

Relative attraction of male *B. latifrons* was assessed to alpha-ionol, alpha-ionol + cade oil, alpha-ionol + eugenol, dihydroeugenol, isoeugenol, and methyl-isoeugenol. All lures were applied to 3.8 × 1.0 cm (diam) dental wicks (Livingstone Int., Rosebury, New South Wales, Australia). 3.0 mL was applied for each single component lure (spread over two wicks), while dual component lures were dosed with 2.0 mL of alpha-ionol on one wick + 1.0 mL of the 2nd component on a 2nd wick. Wicks were enclosed in plastic baskets (with each component of the dual component lures held in separate baskets). Baskets were then slid on to the hanger of a Jackson trap (Scentry Biologicals, Inc., Billings, MT, USA). Attracted flies were immobilized on the inserted sticky card, with no toxicant used with the trap. The trial was conducted over a period of seven weeks, with freshly-treated wicks and new Jackson traps used at the start of each week. Traps were deployed on 10 October 2017, with the test terminated on 28 November 2017. Because of a flash flood warning, servicing of traps in Week 2 was done one day late (8 day service), while servicing of traps in Week 3 was done one day early (6 day trap service). Because of staff illness, deployment of traps for Week 5 was done one week after the servicing of traps in Week 4. For each week, one trap of each treatment was deployed in each of six turkeyberry patches (blocks). Distance between traps within a patch was at least 5.0 m, with trap positions fixed throughout the trial. The 5.0 m spacing permitted inclusion of all traps within a block to be contained within a given turkeyberry patch, and is far enough apart that there should be no interference effect as 3.0 m separation between cuelure and methyl eugenol traps has not been found to result in an interference effect [25] and alpha-ionol + cade oil is not as strong an attractant as either cuelure or methyl eugenol [20]. Distance between the midpoints of adjacent patches averaged 62.4 m (range: 32.1–97.2 m). The relative position of each trap in a patch was randomized, with new position randomizations made each week in such a manner that, by the end of the test, each trap in a given patch had been in each trap position once.

### 2.4. Statistical Analyses

*Bactrocera latifrons* trap catch and *B. dorsalis* trap catch was averaged by treatment each week. Using JMP 11.2.0, a two-way analysis of variance (ANOVA) (treatment, block) was performed, for each tephritid fruit fly species separately, on square root transformed (square root [x + 0.5]) catch values, with Tukey HSD used for means separation [26]. Because of potential flash flood conditions, trap servicing had to be delayed one day for Week 2. The eight day trap catch results in Week 2 and six day trap catch results for Week 3 were adjusted to seven day catches based on the average catch per day results. All other weeks were seven day catches.

## 3. Results

**Figure 1:**
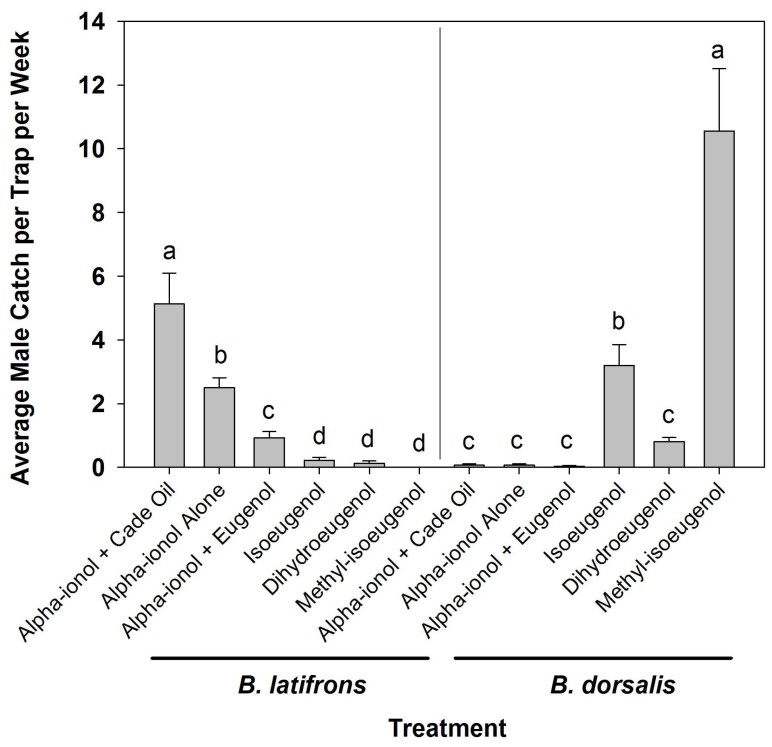
Average male *B. latifrons* catch (left) and *B. dorsalis* catch (right) per trap per week by treatment. Columns with the same letters are not significantly different at the α = 0.05 level. Analysis of variance (ANOVA) was run separately for *B. latifrons* and *B. dorsalis* catches.

There were significant differences overall in male *B. latifrons* catch (F = 32.31; df = 11, 168; *p* < 0.0001) among treatments (*p* < 0.0001), blocks (*p* = 0.0017) and the treatment-block interaction (*p* = 0.0004). There were also significant differences overall in male *B. dorsalis* catch (F = 38.79; df = 11, 168; *p* < 0.0001), with significant differences among treatments (*p* < 0.0001), but not among blocks (*p* = 0.0560) or in the treatment-block interaction (*p* = 0.6764). For *B. latifrons*, catch in traps baited with alpha-ionol + cade oil was significantly greater than in traps baited with alpha-ionol alone which was significantly greater than in traps baited with alpha-ionol + eugenol which was significantly greater than in traps baited with dihydroeugenol, isoeugenol or methyl-isoeugenol. There was no significant difference in catch among traps baited with dihydroeugenol, isoeugenol or methyl isoeugenol (see Figure 1). No male *B. latifrons* were caught in any traps baited with methyl-isoeugenol throughout the entire test, though two female *B. latifrons* were caught at this lure.

For *B. dorsalis*, catch in traps baited with methyl-isoeugenol was significantly greater than catch in traps baited with isoeugenol which was significantly greater than catch in traps baited with dihydroeugenol, alpha-ionol alone, alpha-ionol + cade oil or alpha-ionol + eugenol. There was no significant difference in catch among traps baited with dihydroeugenol, alpha-ionol alone, alpha-ionol + cade oil or alpha-ionol + eugenol (see Figure 1). *Bactrocera dorsalis* was recovered in all six treatments, but never more than one per trap in any of the treatments that included alpha-ionol. Actual and adjusted *B. dorsalis* trap catch data is available in Supplementary Materials File S1.

## 4. Discussion

*Bactrocera latifrons* was recovered in traps baited with two of the three eugenol analogs tested (dihydroeugenol and isoeugenol), but catch was significantly lower than catch in traps baited with alpha-ionol alone or alpha-ionol + cade oil (Figure 1). *Bactrocera latifrons* catch in traps baited with alpha-ionol + eugenol was significantly less than catch in traps baited with either alpha-ionol alone or alpha-ionol + cade oil. Similarly, an earlier trial with wild flies also showed that catch in traps baited with alpha-ionol + eugenol was significantly less than catch in traps baited with alpha-ionol + cade oil on three of four weeks at two sites on Maui, Hawaii [18]. However, that trial did not include an “alpha-ionol only” treatment so comparison of trap response to “alpha-ionol only” baited traps was not possible. Conversely, earlier field tests with sterile *B. latifrons* had shown catch in traps baited with alpha-ionol + eugenol to not differ significantly from catch in traps baited with alpha-ionol + cade oil with relative catch in the alpha-ionol + eugenol traps increasing as the eugenol proportion increased from 50:1 to 2:1 (alpha-ionol: eugenol). In the test at the 2:1 ratio, catch was actually numerically higher in traps baited with alpha-ionol + eugenol than in traps baited with alpha-ionol + cade oil [18]. That is the same ratio, though with only half the compound doses, as used in the present trial. This may be an indication that sterile and/or colony flies may have slightly different lure responses than wild flies or may just represent sample variation where alpha-ionol/eugenol response may typically be somewhat less than alpha-ionol/cade oil response, but may range from approximately the same to significantly less.

Of the three eugenol analogs tested here, isoeugenol had previously been tested in olfactometer screening (using about 0.02 mL, mixed in water, ethanol, and polyethylene glycol) as a potential *B. latifrons* attractant, with male catches at isoeugenol baited traps 23.1% of catches at alpha-ionone (structurally very similar to alpha-ionol) and 68.5% of catches at methyl eugenol [19]. Both isoeugenol and dihydroeugenol were later identified as constituents of cade oil that were potentially synergistic with alpha-ionol in attracting male *B. latifrons*. In tests of isoeugenol and dihydroeugenol as potential alpha-ionol synergists, synergistic enhancement of catch in traps baited with alpha-ionol typically more closely approached the synergistic enhancement provided by cade oil when the relative concentration of the potential synergist was increased (up to about 1:1) [18]. Neither of these studies, though, tested isoeugenol or dihydroeugenol at the higher doses used in trapping programs (3 mL undiluted) in the absence of alpha-ionol. Our present study showed that the higher lures dose (3 mL pure lure) did not greatly increase the attraction of *B. latifrons*, although there are studies that suggest this may be expected. Methyl eugenol is a phenylpropanoid with a high rate of volatilization of 26.4 mg/day at 24 °C [27] and consequently small doses of 0.02 mL [19] would evaporate within a day. The eugenol analogs used herein are also phenylpropanoids with a similar chemical structure to methyl eugenol, and presumably also have a high rate of volatilization.

Isoeugenol, methyl-isoeugenol and dihydroeugenol have been tested in several other countries and found to attract non-responsive species or more strongly attract some methyl eugenol and cuelure responsive species. This suggested that they may have potential as male lures for *B. latifrons.* In Australia, isoeugenol was more attractive to the cuelure-responsive pest species *B. kraussi* (Hardy), all three lures were more attractive to the methyl eugenol-responsive species *B. yorkensis* Drew & Hancock and attracted four non-responsive species (*B. barringtoniae* (Tryon), *B. bidentata* (May), *B. halfordiae* (Tryon) and *B. murrayi* (Perkins)) [10]. In Papua New Guinea, methyl-isoeugenol and isoeugenol were weakly attractive to the minor pest *B. obliqua* (Malloch) [9]. In Bangladesh, methyl-isoeugenol was almost 50× more attractive to the methyl eugenol-responsive cucurbit flower pest *Zeugodacus diversus* (Coquillett) than methyl eugenol [28]. These lures were also recently found to be more attractive than the known lures to two pest species in the Pacific: the methyl eugenol-responsive Pacific fruit fly *B. xanthodes* (Broun) [29] and the cuelure-responsive *B. curvipennis* (Froggatt) [30]. As these lures have proven to be attractive to a number of species it was considered possible that *B. latifrons* may also respond, particularly given its previous responses to low doses of isoeugenol and the structurally similar methyl eugenol [18,19].

While *B. latifrons* showed a response to methyl eugenol in an olfactometer test [19] and in a field test with released sterile flies [18], its response is so weak that methyl eugenol is not a very effective detection tool. As for other novel male lures it also has shown no response to zingerone in preliminary laboratory and field tests in Hawaii [31]. In laboratory tests, 3-oxygenated derivatives of alpha-ionol and alpha-ionone have been shown to be more attractive to *B. latifrons* [32], but had little attraction in the field, likely due to low volatility [33]. Protein based lures are also less attractive to *B. latifrons* than alpha-ionol plus cade oil [20,22]. The results from this study, along with earlier work, show that alpha-ionol and cade oil continue to be the best attractants for *B. latifrons.*

## 5. Conclusions

Isoeugenol, dihydroeugenol, methyl-isoeugenol, alpha-ionol and alpha-ionol + eugenol were field tested as male attractants for *B. latifrons* in comparison to the standard lure alpha-ionol + cade oil. Alpha-ionol + cade oil was significantly more attractive than all the other lures, demonstrating that it continues to be the most effective attractant for male *B. latifrons.*

## Supplementary Materials

The following is available online at http://www.mdpi.com/2075-4450/9/2/50/s1. File S1: McQuateRoyerSylva-Bactrocera_latifrons_and_Bactrocera_dorsalis-TrapCatchData.
